# Feasibility of the annulus fibrosus repair with in situ gelating hydrogels – A biomechanical study

**DOI:** 10.1371/journal.pone.0208460

**Published:** 2018-12-06

**Authors:** Anne-Gita Scheibler, Tobias Götschi, Jonas Widmer, Claude Holenstein, Thomas Steffen, Roland S. Camenzind, Jess G. Snedeker, Mazda Farshad

**Affiliations:** 1 Department of Orthopedics, Balgrist University Hospital, Zurich, Switzerland; 2 Institute for Biomechanics, ETH Zurich, Zurich, Switzerland; 3 Musculoskeletal Research Unit (MSRU), Center for Applied Biotechnology & Molecular Medicine (CABMM), University of Zurich, Zurich, Switzerland; National University of Ireland - Galway, IRELAND

## Abstract

The surgical standard of care for lumbar discectomy leaves the annulus fibrosus (AF) defect unrepaired, despite considerable risk for a recurrent herniation. Identification of a viable defect repair strategy has until now been elusive. The scope of this ex vivo biomechanical study was to evaluate crosslinking hydrogels as potentially promising AF defect sealants, and provide a baseline for their use in combination with collagen scaffolds that restore disc volume. This study directly compared genipin crosslinked fibrin hydrogel (FibGen) as a promising preclinical candidate against a clinically available adhesive composed of glutaraldehyde and albumin (BioGlue). Forty-two bovine coccygeal functional spine units (FSU) were randomly allocated into four groups, namely untreated (control, n = 12), repaired with either one of the tested hydrogels (BioGlue, n = 12; FibGen, n = 12), or FibGen used in combination with a collagen hydrogel scaffold (FibGen+Scaffold, n = 6). All specimens underwent a moderate mechanical testing protocol in intact, injured and repaired states. After completion of the moderate testing protocol, the samples underwent a ramp-to-failure test. Lumbar discectomy destabilized the FSU as quantified by increased torsional range of motion (28.0° (19.1, 45.1) vs. 41.39° (27.3, 84.9), p<0.001), torsional neutral zone (3.1° (1.2, 7.7) vs. 4.8° (2.1, 12.1), Z = -3.49, p < 0.001), hysteresis(24.4 J (12.8, 76.0) vs. 27.6 J (16.4, 54.4), Z = -2.61, p = 0.009), with loss of both disc height (7.0 mm (5.0, 10.5) vs 6.1 mm (4.0, 9.3), Z = -5.16, p < 0.001) and torsional stiffness (0.76 Nmdeg^-1^ (0.38, 1.07) vs. 0.66 Nmdeg^-1^ (0.38, 0.97), Z = -3.98, p < 0.001). Most FibGen repaired AF endured the entire testing procedure whereas only a minority of BioGlue repaired AF and all FibGen+Scaffold repaired AF failed (6/10 vs. 3/12 vs. 0/6 respectively, p = 0.041). Both BioGlue and FibGen+Scaffold repaired AF partially restored disc height (0.47 mm (0.07, 2.41), p = 0.048 and 1.52 mm (0.41, 2.57), p = 0.021 respectively) compared to sham treatment (0.08 mm (-0.63, 0.88)) whereas FibGen-only repaired AF had no such effect (0.04 mm (-0.73, 1.13), U = 48.0, p = 1). The AF injury model demonstrated considerable change of FSU mechanics that could be partially restored by use of an AF sealant. While inclusion of a volumetric collagen scaffold led to repair failure, use of FibGen alone demonstrated clinically relevant promise for prevention of mechanical reherniation, outperforming an FDA approved sealant in this ex vivo test series.

## Introduction

Intervertebral disc herniation is one of the most frequent causes of low back pain [1–3]. Here, defects within the annulus fibrosus (AF) allow expulsion of nucleus pulposus (NP) material, which in turn can provoke nerve irritation [1–3]. Lumbar discectomy has emerged as the most commonly applied and effective method for surgical treatment of intervertebral disc herniation [4,5]. Current practice involves removal of herniated material through the AF defect or through a surgical incision, leaving an unrepaired defect within the AF. Recurrent disc herniation remains a considerable risk [6,7]. Moreover, discectomy is associated with loss of disc height, torsional stiffness and NP fluid pressurization [8]. These changes of intervertebral disc (IVD) biomechanics yield decreased joint stability and have been linked to accelerated IVD and facet joint degeneration [8–10]. To date, no reliably effective method for AF repair has been established.

In view of this large clinical need, various therapeutic strategies for AF repair have been suggested and pursued. Ideally, a repair strategy should be easily applicable, restore primary biomechanical properties, withstand in-vivo loading during daily activities, and ultimately mitigate risk of reherniation [11]. A variety of methods have been developed to meet these requirements, yet have been met with limited success. For instance, modified suturing techniques have failed to restore biomechanical properties as they do not substitute the loss of NP volume [12]. Although AF closure devices have shown promising results in vitro, longer-term performance in large animal in vivo testing has been unsatisfactory [13], potentially due to mismatched biomaterial-tissue elasticity [11].

Preclinical research and development efforts to move the field forward have recently focused on the application of hydrogels offering tunable mechanical properties, with capacity for *in situ* gelation, and the possibility to seal tissues by nature of their inherent adhesive properties. For instance genipin has emerged as a naturally occurring low-toxicity crosslinking agent with potential for stabilizing both synthetic polymers and biological tissues [14,15]. Specifically regarding AF repair, early results using genipin crosslinked fibrin hydrogel (FibGen) have been promising in partially restoring biomechanics of the functional spine unit (FSU), however failing under higher ranges of physiological loads [16–19] [19]. Bioglue is a commercially available (FDA approved) hydrogel composed of glutaraldehyde and albumin [20,21]. It provides fast gelation time and good biocompatibility as the glutaraldehyde is mixed completely with the albumin within the application tube [22]. BioGlue has been reported to be an effective tissue sealant in dural, vascular, intestinal and pulmonary surgeries [21,23] with high adhesive strength and pressure retention capabilities [24]. However, no study has yet evaluated the potential of BioGlue for use in AF closure.

The scope of the present *ex vivo* study was to compare and evaluate two crosslinking hydrogels, FibGen and BioGlue, as possible AF sealants for prevention of recurrent herniation (1) and restoration of biomechanical properties to the intact state (2). We then evaluated the most promising of these hydrogels to be used in combination with a collagen scaffold to account for loss of disc height (3).Collagen scaffolds may enable a favorable environment for secondary tissue ingrowth and repair. Such composite repair approaches that combine hydrogels with scaffolds have been suggested as promising [19] in compensating NP-volume loss and facilitating tissue remodeling [25].

## Material and methods

### Dissection, preparation and storage

Twenty-one frozen, young adult bovine coccygeal spine segments (coccygeal vertebrae 1–4) were isolated from animals sacrificed for food purposes (Metzgerei Angst AG, Zurich, Switzerland). Hence no ethical approval was required according to local regulation. Compared to human IVDs bovine coccygeal IVDs show similar cross-sectional area and material properties [26]. Moreover, they are easily available and relatively uniform and therefore provide minimal interspecimen variability. Frozen specimens were thawed and two functional spine units (FSU) of each bovine spine (coccygeal vertebrae 1–2, coccyceal vertebrae 3–4) were dissected. A total of 42 FSUs were potted in a beracryl-monomer mass. During the potting procedure the specimens were wrapped in a PBS-soaked cloth to prevent dehydration. The diameter of the intervertebral discs was measured using a caliper. All the potted specimens were x-rayed in order to measure disc height. They were then stored in the freezer (-20°C) until final experimental use with no additional freeze-thaw-cycles taking place [27].

### Biomechanical testing

**Fig 1:**
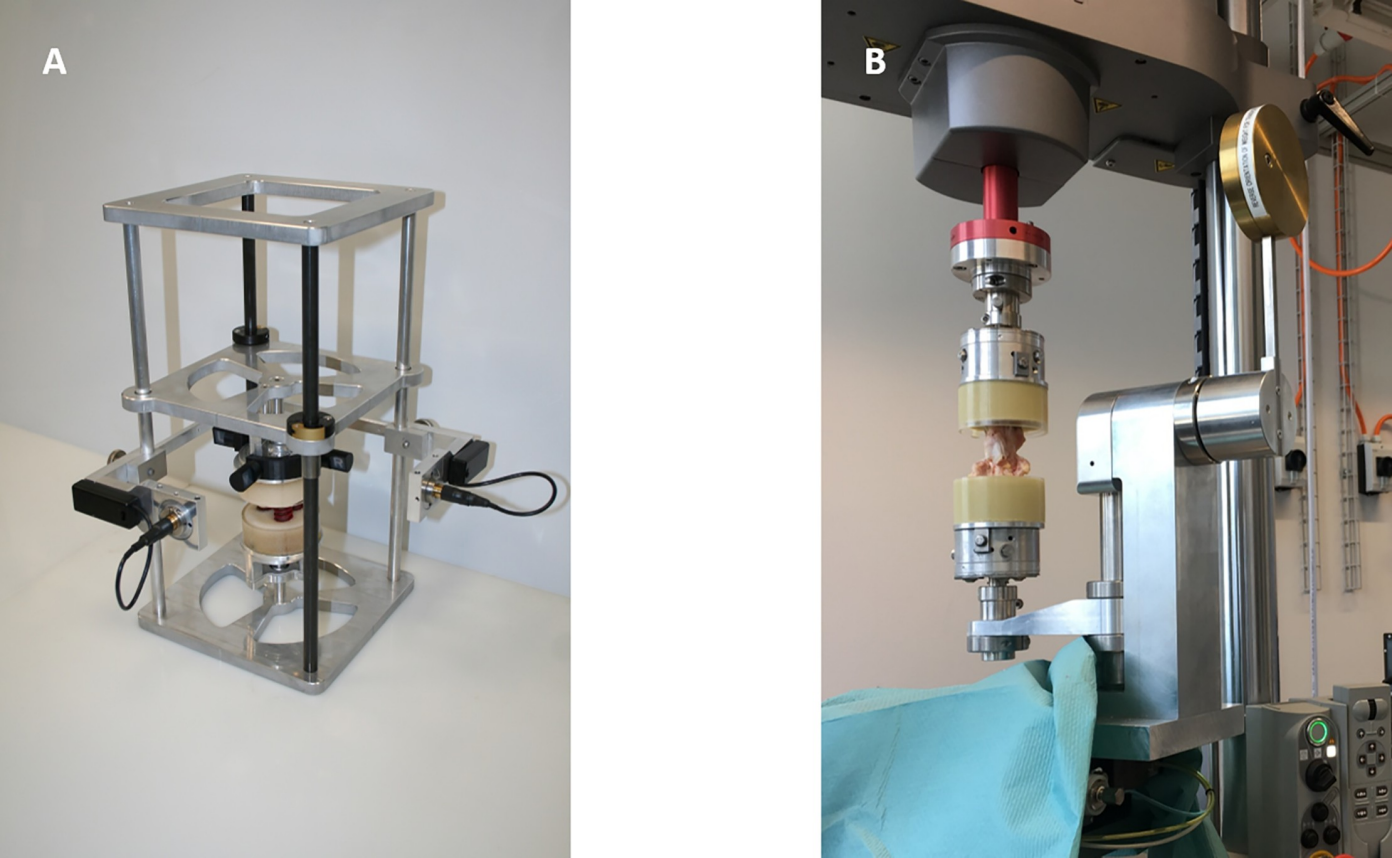
Test setup for biomechanical testing of the bovine functional spine units. (A) Calibration frame for laser-guided specimen orientation. The degrees of freedom of the specimen clamping system allow independent rotation of both spine segments for specimen alignment into neutral position. (B) Biaxial testing machine with custom-built fixation setup and aligned test specimen.

The specimens were thawed and rehydrated for 3h at room temperature in PBS (containing 3% Penicillin/Streptomycin (Biowest) and 1.5% Fungizone (Gibco^TM^) to ensure a uniform state of initial hydration. For each coccygeal spine segment, the bony endplates were minimally embedded in cylindrical methylmetacrylate blocks (Beracryl D28, Suter Kunststoffe AG, Fraubrunnen), poured in 80mm x 40mm (d x h) thin-walled acrylic tubes. Both poured blocks then were fitted within alignment plates that engaged with the freely swiveling mounting platforms of a custom calibration frame (Fig 1A). Guided with projected laser lines, the specimen was visually centered and aligned to its anatomical planes (vertical midsagittal plane, horizontal disc midplane), with the alignment plates being then fixed for the duration of the experiment by three threaded bolts. The above procedure resulted in a highly controlled centering and alignment of the specimens, even when the molded acrylic blocks were themselves not centered and/or misaligned.

The biomechanical testing protocols were performed on a biaxial linear-torsion electromechanical dynamic testing system (ElectroPlus^TM^ E3000, Instron Corp., Canton, MA, USA), that was fitted with a custom-built setup to allow for additional passive axes to be controlled. The specimen was mounted, pre-calibrated to its anatomical planes, between the two precisely vertically aligned pivot locks (using the XY sliding table with the shaft collars fastened, mono-arm swing fixed in its lower most position). Both pivot locks then were tightened (Fig 1B), resulting in a force free mounted specimen that was precisely aligned to its anatomical planes.

**Fig 2:**
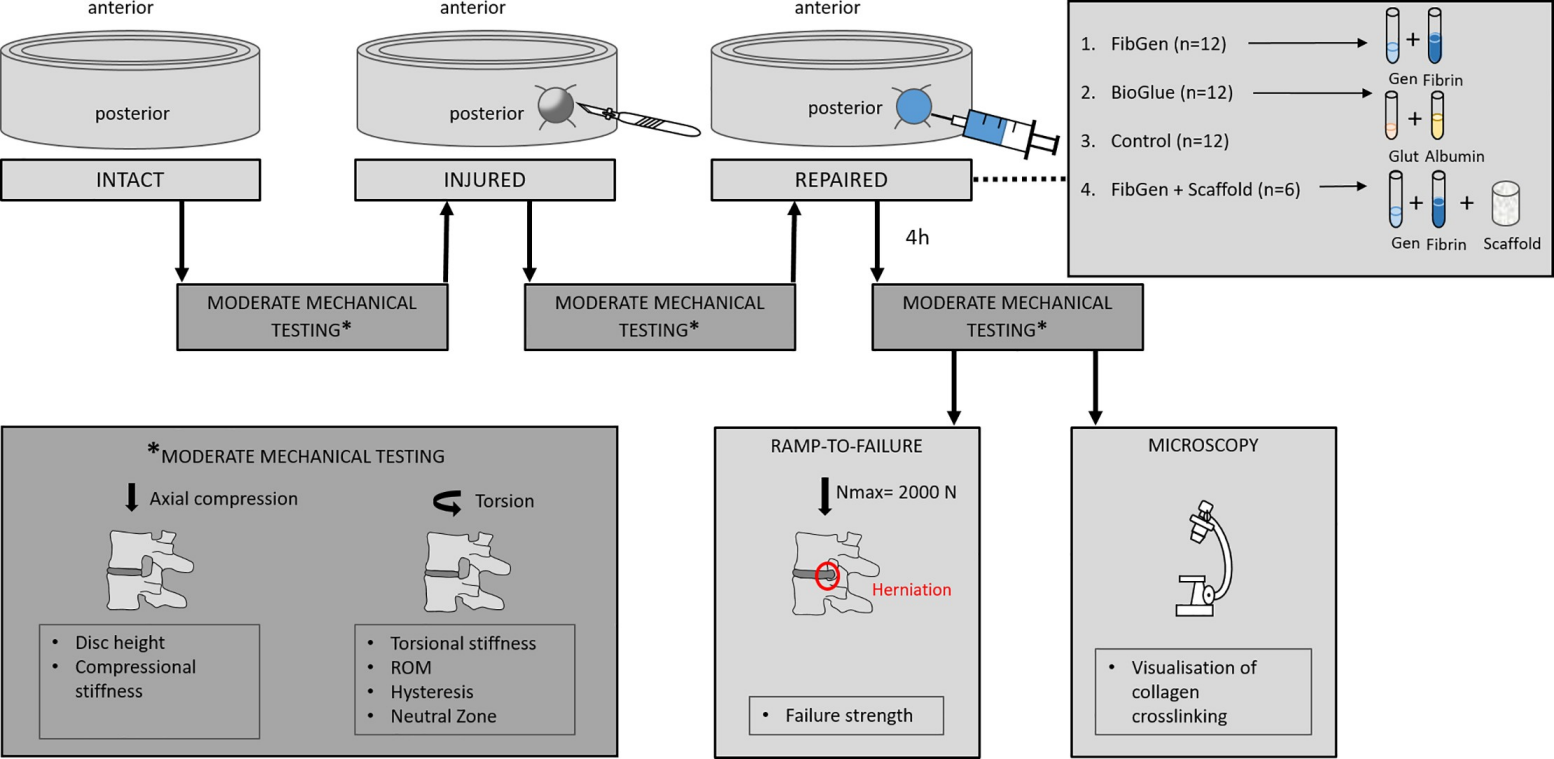
Experimental methodology. AF injury was induced by means of a cruciate incision at the dorsolateral aspect and removal of about 25% (200 mg) of the nucleus pulposus. For defect repair the two components of the hydrogels were mixed and immediately applied (0.2 ml) allowing gelation to take place *in situ*. Abbreviations: Gen = Genipin; Glut = Glutaraldehyde.

In order to obtain a reproducible injury and repair procedure, a single surgeon performed all the injuries and repairs. Each specimen was sequentially tested in intact, injured and repaired state as visualized in Fig 2. To reproduce anatomical positioning (i.e., aligned and centered force-free mounting) of the specimen for each test state, only the pivot locks were loosened for removal of the specimen, then force-free re-tightened upon re-mounting. The alignment plates were not re-adjusted.

### Moderate mechanical testing and injury

The moderate mechanical testing protocol was comprised of axial compression testing and torsion testing. 26 cycles at a linear travel profile with axial stress from 0 to 0.5 MPa at a crosshead speed of 2 mm/min were performed for compressive testing. Throughout testing, axial rotation of the pivot was unconstrained. Except for the calculation of disc height, the 26^th^ cycle was used for calculation of mechanical properties with the purpose of the preceding 25 cycles to reach stress-strain steady-state [28]. A limit of 0.5 MPa resembles the estimated axial stress of upright standing [19]. For torsional testing, both pivot locks were blocked for rotation. A total of three axial torsion cycles at 1°/s constant velocity in both directions was applied. The compressive load (vertical actuator) was adjusted at the onset to read zero, then kept at a fixed height. Torque limits were preset at ±7.5 Nm [29]. Again all but the last cycle were performed to minimize visco-elastic effects [30]. Limits of 7.5 Nm torque were deemed the appropriate maximum torque not resulting in damage to the FSU [31].

After completion of the first test, a cruciate incision was created at the dorsolateral aspect of the AF using a #11 scalpel blade with a depth of approximately 8mm. Then about 25% (200mg) of the NP were removed by means of an abrasor (210 ± 21 mg) [19]. Subsequently the moderate mechanical testing protocol was repeated. Immediately after mechanical testing was completed samples were wrapped with PBS-soaked cloth between testing.

### Annulus fibrosus repair

**Fig 3:**
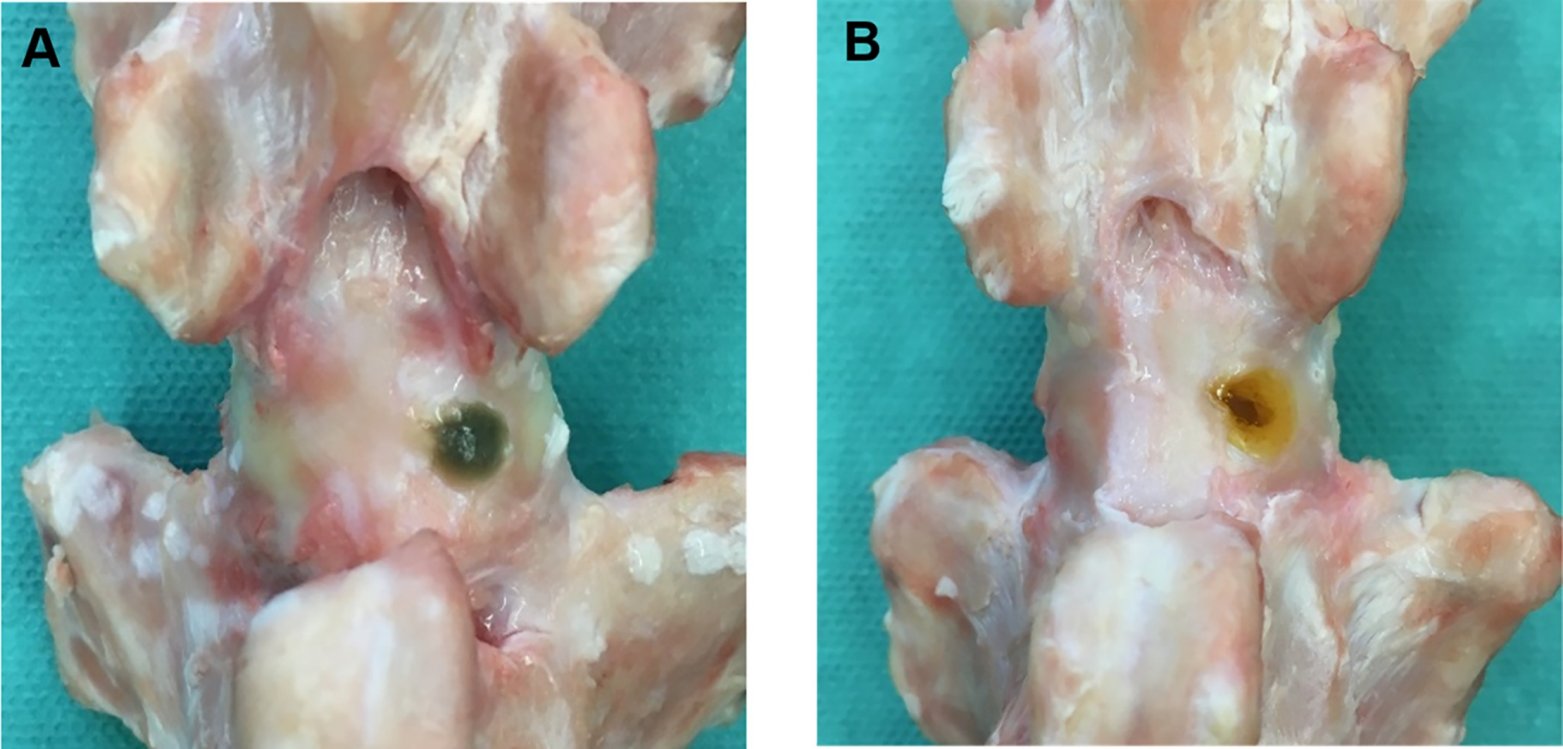
Dorsal view of a functional spine unit 4 hours after repair. (A) FibGen repair (B) BioGlue repair.

42 FSUs were randomly distributed into four groups: Control (N = 12), BioGlue (N = 12), FibGen (N = 12), FibGen + Collagen Scaffold (N = 6). During testing 4 (2 Control, 2 FibGen) FSU samples had to be excluded due to incorrect size (n = 1), critical laceration during dissection (n = 1) and unstable vertebrae potting (n = 2). The control group was left unrepaired. In the BioGlue and FibGen group, the defect was repaired with the corresponding hydrogel (Fig 3). The two components of the hydrogel were premixed and immediately applied to the injury using a pipette (0.2 ml).

FibGen was prepared as described earlier [18]. Briefly 56 U/ml of thrombin was mixed with 6 mg/ml genipin (400 mg/mL dissolved in DMSO) and PBS. For the second component, 140 mg/ml fibrinogen was dissolved in PBS and then mixed in the ratio 4:1 with the first component during application. For the preparation of BioGlue 45% w/v of serum albumin was mixed with PBS for the first component and 10% w/v glutaraldehyde was mixed with PBS for the second component [32]. For scaffold-augmented repair a porcine, porous, volume-stable collagen matrix (Geistlich Fibro-Gide, Geistlich Pharma AG, Wolhusen, Switzerland) was cut into a cylindrical shape with dimensions of 5 and 7 mm in diameter and length respectively. The collagen scaffold was presoaked in hydrogel and inserted immediately. Subsequently 0.1ml FibGen was applied for AF closure.

After repair, the samples were kept at room temperature wrapped in PBS-soaked cloth. A total of 4 hours for repair were set to represent the time from surgery until the patient first moves.

### Ramp-to-failure testing

After completion of the moderate mechanical testing protocol, the samples underwent a ramp-to-failure test at a crosshead speed of 2 mm/min. Axial compression was increased up to a maximum of 2000 N or until herniation occurred. Two examiners observed and recorded herniation.

### Data analysis of mechanical testing

Parameters evaluated for compressive testing included compressive stiffness and disc height at each repeated test. Compressive stiffness was defined as the slope of the linear fit to the last 20% position of the force-position signal [19,33]. Initial disc height was measured on plain radiographs by averaging the shortest distances between endplates on antero-posterior and lateral view. Disc height loss was then calculated as the change in height from intact state over the course of testing at 0.5 MPa of the first compressive cycle. Torsional testing included the parameters torsional range of motion (ROM), neutral zone, linear region stiffness and hysteresis. ROM was defined as the total rotation within the torque targets. Neutral zone was defined as the difference in angulation between the two phases of motion at zero torque [28]. Linear region stiffness was calculated equivalently to the calculation of compressive stiffness. Hysteresis describes energy dissipation during the evaluated torsion cycle and was defined as the area enclosed by the torque-angulation curve [28]. Calculation of mechanical parameters was performed with MATLAB (Release 2017b, The MathWorks, Inc., Natick, Massachusetts, United States.)

### Statistical analysis

Visual data inspection and Kolmogorov-Smirnov testing revealed various factor-levels to be distributed non-normally. Consequently, non-parametric means for inference testing were employed. Differences in mechanical parameters between intact and injured state were investigated using Wilcoxon signed-rank test. Differences between repair type on biomechanical restoration capacity was investigated on relative changes in biomechanical parameters from injured to repaired state using univariate Kruskal-Wallis analysis of variance (ANOVA). Significant factors were further investigated by pairwise comparison of each repair to the control group with Bonferroni-correction of p-values. Fisher’s exact tests were then applied to examine differences in failure frequency between the repair techniques at the three steps of the testing procedure. Repair strength was investigated by comparing maximum compressive force achieved without herniation between the repair techniques using Mann-Whitney U-testing. If not otherwise specified, group characteristics are presented with median and range or in absolute frequencies as applicable. P < 0.05 was set for statistical significance. Data analysis was performed using SPSS (IBM Corp. Released 2017. IBM SPSS Statistics for Windows, Version 25.0. Armonk, NY: IBM Corp.).

### Microscopy

In order to qualitatively assess the spatial and temporal progression of genipin-induced crosslinking, the tissue was visualized using a variant of our previously developed method based on fluorescence microscopy [34]. Confocal fluorescence imaging was performed using an inverted spinning disk confocal microscope (iMic, Fei Munich GmbH) using a 4x (0.16 NA) objective (OlympusTM U Plan S-Apo; Thermo Fisher) and a Hamamatsu Orca-flash 4.0 V2 Digital CMOS camera C11440-22CU (Hamamatsu Photonics KK, Hamamatsu City, Japan). One FSU sample repaired using FibGen was cut in the transversal plane parallel to the endplates through the center of the repair. The sample was mounted on an in-house built flow chamber to ensure constant circulation of fresh 1x PBS during imaging. Making use of their autofluorescent behavior, collagen type I was made visible with a laser excitation wavelength of 405 nm [35] and the genipin crosslinks with an excitation wavelength of 560 nm [34]. In order to cover the whole sample, 330 single z-stacks (22x15) were acquired with an overlap of 10% for 7 different time points starting 90 min after repair, automated by the built-in “live Acquisition” software (Fei Munich GmbH, Munich, Germany). The images were then stitched and a maximum intensity projection was performed using the “Offline Analysis” software (Fei Munich GmbH, Munich, Germany), resulting in an approx. 280 Megapixel images. Final image processing was done using ImageJ [36].

## Results

### Moderate mechanical testing

Injury to the AF resulted in a significant increase in torsional ROM (28.0° (19.1, 45.1) vs. 41.4° (27.3, 84.9), Z = -4.11, p < 0.001) torsional neutral zone (3.1° (1.2, 7.7) vs. 4.8° (2.1, 12.1), Z = -3.49, p < 0.001), compressive stiffness (221 Nmm^-1^ (146, 306) vs. 226 Nmm^-1^ (143, 331), Z = -2.42, p = 0.015) and hysteresis (24.4 J (12.8, 76.0) vs. 27.6 J (16.4, 54.4), Z = -2.61, p = 0.009) whereas a significant reduction in disc height (7.0 mm (5.0, 10.5) vs 6.1 mm (4.0, 9.3), Z = -5.16, p < 0.001) and torsional stiffness (0.76 Nmdeg^-1^ (0.38, 1.07) vs. 0.66 Nmdeg^-1^ (0.38, 0.97), Z = -3.98, p < 0.001) was observed.

**Fig 4:**
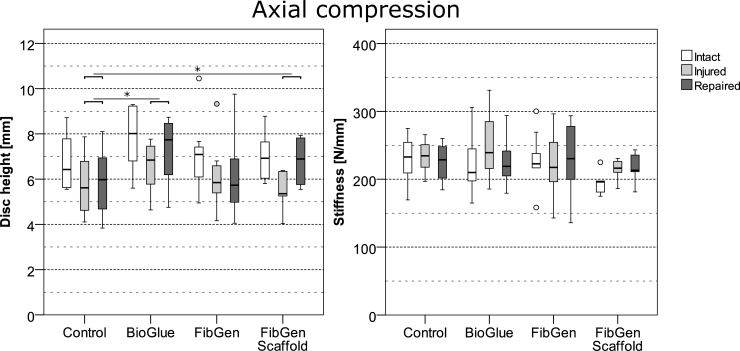
Mechanical properties of the three tested repairs in axial compression in intact state, after injury and after repair. Significant differences in disc height gain from injured to repaired state are indicated with an asterisk.

**Fig 5:**
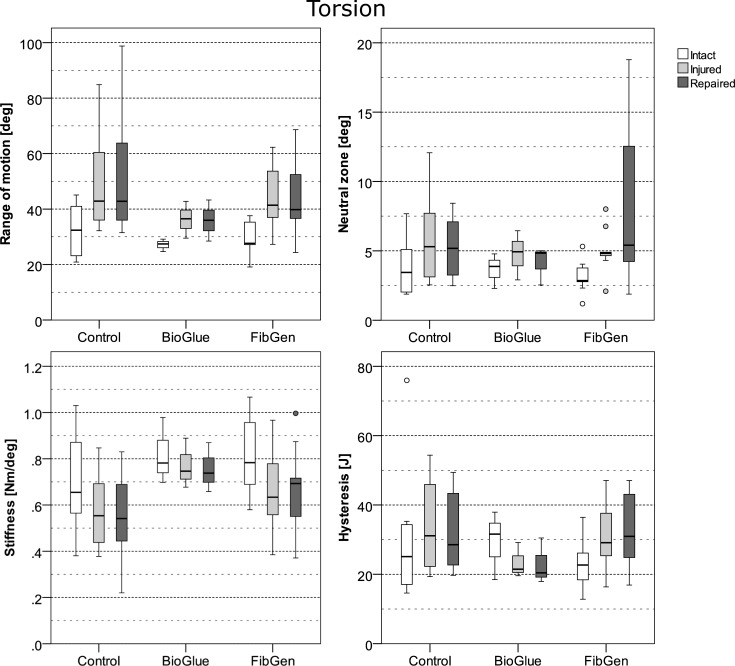
Mechanical properties of the three tested repairs in axial torsion in intact state, after injury and after repair.

The applied type of repair had a statistically significant effect on disc height (χ2(3) = 13.01, p = 0.005) and compressive stiffness (χ2(3) = 9.65, p = 0.022) but not on torsional ROM (χ2(2) = 2.15, p = 0.342), torsional stiffness (χ2(2) = 0.02, p = 0.990), torsional neutral zone (χ2(2) = 4.432, p = 0.109) and hysteresis (χ2(2) = 0.985, p = 0.611) determined by Kruskal-Wallis ANOVA testing. Post-hoc pairwise comparisons of the significant outcome variables disc height and compressive stiffness revealed a significant difference in disc height gain of BioGlue (0.47 mm (0.07, 2.41), U = 18.0, p = 0.048) and FibGen + Scaffold (1.52 mm (0.41, 2.57), U = 3.0, p = 0.021) but not FibGen (0.04 mm (-0.73, 1.13), U = 48.0, p = 1) repaired AF compared to the control group (0.08 mm (-0.63, 0.88)) after Bonferroni-correction. Pairwise comparisons of relative change in compressive stiffness after the repair revealed nonsignificant differences of BioGlue (-8% (-23, 5), U = 34.0, p = 0.678), FibGen+Scaffold (1% (-3, 6), U = 13.0, p = 0.426) and FibGen (-1% (-9, 19), U = 48.0, p = 1) repaired AF compared to the control group (Figs 4 and 5).

### Repair failure

**Fig 6:**
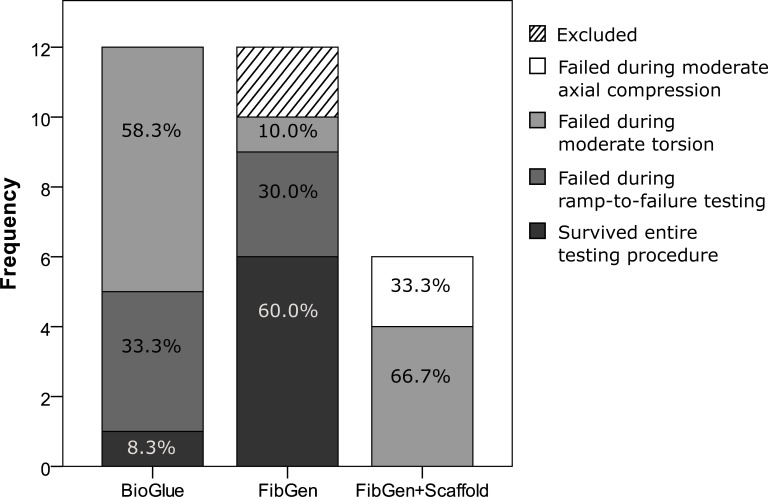
Failure frequencies and percentages of the tested repairs during the entire testing procedure.

Two FibGen+Scaffold samples failed during moderate axial testing whereas all BioGlue and FibGen repairs survived this first step of the testing procedure (p = 0.021). Seven (58.3%) BioGlue samples failed during moderate torsional testing whereas only one FibGen sample (10%) but all four remaining FibGen+Scaffold (66.7%) samples failed (p < 0.001). Of the remaining 5 BioGlue samples only one (8.3%) in five but six (60.0%) in nine FibGen samples survived ramp-to-failure testing (p = 0.003) (Fig 6). Of the samples surviving moderate testing median ultimate failure strength was 1240 N (490, 2000) and 2000 N (1200, 2000) for BioGlue and FibGen respectively (U = 8.50, p = 0.046).

### Microscopy

**Fig 7:**
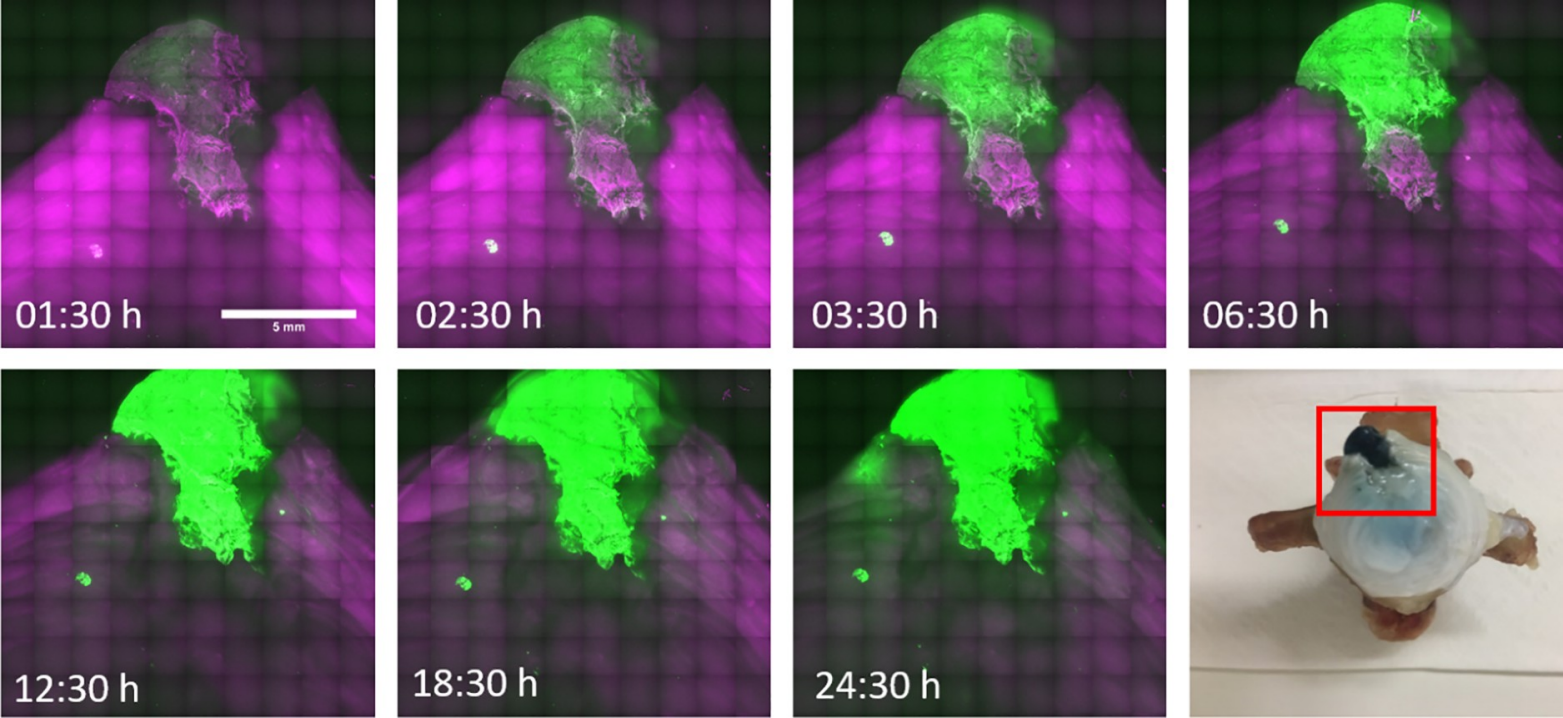
Time-lapse confocal microscopy of a FibGen repaired annulus fibrosus. Gradual crosslinking of the hydrogel and the adjacent tissue within 24 hours upon application was shown. Collagen-specific fluorescence in purple and genipin-induced crosslinking in green. Bottom right: Macroscopic view of the repaired AF with the FibGen repair in blue and the window size for microscopy in red.

Genipin-induced collagen crosslinks emit fluorescent light at 645 nm when excited at 590 nm [34]. By exploiting this property, fluorescence imaging of a FibGen repaired AF visualized by confocal microscopy revealed gradual crosslinking of the hydrogel evident by an increase in fluorescence emission over time (Fig 7). The fluorescent signal was strongest in the area containing FibGen and the gradual increase of the fluorescence in the surrounding area indicates diffusion of the genipin crosslinks to the adjacent tissue.

## Discussion

The present work is motivated by the clinical need for an AF repair strategy that can reduce risk of recurrent IVD herniation after partial discectomy. A successful AF repair ideally should prevent reherniation (1), restore biomechanical properties to intact state (2) and offer good biocompatibility [11]. From this perspective, we compared two different low-toxicity crosslinking hydrogels, FibGen and BioGlue, with regard to their potential utility for AF repair. The most clinically relevant finding of this comparison was a biomechanical superiority of FibGen repair evidenced by a two-fold reduction in reherniation rate. FibGen used in conjunction with a hydrogel collagen scaffold (3) indicated that this composite approach could in fact restore disc height and initial biomechanical properties, but with failure strengths that were too low to be clinically viable.

Consistent with the lower *in vitro* post-treatment herniation rate of the FibGen treated specimens, this group also showed a higher failure strength in ramp-to-failure testing compared to BioGlue. Most BioGlue repaired AF failed during torsional testing within a physiological torque range whereas all but one FibGen repaired AF endured full torsional testing. These results align with other studies reporting herniation of FibGen treated defects at hyper-physiological rotation angles [19,37]. Macroscopically, repair failure occurred at the interface between BioGlue and native AF tissue, possibly due to material stiffness mismatch and local stress concentrations [38,39]. In contrast, FibGen samples indicated hydrogel cracking to be the primary mode of failure. Generally, FibGen performance was superior in axial compression in terms of herniation prevention compared to BioGlue. Median axial load tolerated by FibGen corresponds to the estimated load exerted on a lumbar FSU when being in a 30° flexed position with straight arms carrying a light weight (~8 kg) [40]. In combination with a collagen scaffold however, only moderate loading was tolerated by the repair with all samples failing during moderate mechanical testing. Samples failing prematurely were excluded from the analysis and therefore no data is available on torsional biomechanics for the composite (FibGen + Scaffold) repair.

Apart from failure strength, the degree to which the different repairs could restore native IVD biomechanics was investigated. Restoration of torsional stiffness has been identified to be the most sensitive benchmark for in vitro testing of AF repair methods [8]. Though not significant, the trends indicated in this study are in agreement with previous FibGen based repairs, which partly restored torsional stiffness [19]. No similar trend was observed for BioGlue repairs. By impairing the structural integrity of the AF torsional ROM and torsional NZ of the FSU are increased [41]. The IVD loses elasticity and the capacity for energy preservation is diminished [41]. Cruciate damage to the AF also resulted in considerable disc height loss. Loss of disc height is associated with progressive IVD and facet joint degeneration, rendering its restoration an essential criterion for an effective disc repair [11]. Long et al. reported partial disc height restoration with FibGen repair yet, our data did not support these findings [19]. FibGen augmentation with a collagen scaffold could achieve a significant gain in disc height, although failing at relatively low compression loads–indicating that volumetric filling of the defect increases the stresses on the construct in a manner that may lead to failure. Despite its poor mechanical performance in the present test series, the favorable environment for regeneration of collagen tissue may warrant further investigation [42,43]. BioGlue repairs also successfully increased post-surgical disc height which we attribute to the very low gelation time reducing the risk for glue dilution.

Recent studies have investigated genipin for its ability to diffuse and mechanically augment tissue by collagen crosslinking [15,34,44]. Popovich and colleagues [41] report a stabilizing effect on FSU biomechanics following simulated decompression surgery when injected with 0.33% genipin solution. Fluorescence-based analysis of the present study confirmed an increase in genipin-induced crosslinking of the hydrogel over time. Moreover, a gradual diffusion of genipin outside the hydrogel and hence crosslinking of the surrounding AF tissue could be demonstrated. This supports the concept that FibGen application, apart from sealing AF defects, may have clinical utility in structurally augmenting collagen tissue of degenerated AF tissue.

Several limitations to the study should be noted. First, the present study was intended to assess only primary stability during early rehabilitation, and the ability of the surgical construct to resist immediate post-operative loads [45]. Clinically relevant, longer-term tissue fatigue will be influenced by remodeling processes and biologic response that can only realistically be assessed through in vivo investigation. Second, the mechanical testing protocol did not include any lateral bending nor flexion or extension and represented a crude approximation of the in vivo mechanical regime of spinal biomechanics. However, recent studies revealed that torsional stiffness is most sensitive to AF injury whereas no change in bending stiffness was observed [8]. Third, the study used bovine AF tissue bearing the potential risk of bias due to interspecies differences in anatomy such as less disc height compared to human IVDs. However, bovine IVD are widely accepted as a suitable model for biomechanical studies of the IVD as they show similar cross-sectional geometry and material properties as human lumbar disks [46,47].

## Conclusion

We introduce a cruciate AF injury model that resulted in considerable change of FSU mechanics. FibGen repair of these defects was superior to BioGlue with respect to reduced rates of reherniation in the employed *in vitro* test setup. Although our findings do suggest that FibGen augmentation in combination with a collagen hydrogel scaffold holds promise to restore disc height and improve initial biomechanical properties, additional work will be required to make this approach sufficiently robust to withstand even moderate postoperative biomechanical loads in the spine. Although considerable preclinical work remains to be done, we conclude that FibGen based repair of AF defects is both a clinically and biomechanically viable approach.

## Supporting information

S1 File Moderate mechanical testing results. Assessed parameters during repeated mechanical testing for both axial compression and axial torsion. Each specimen underwent three tests at intact state after damage and upon repair. Variables ending in “_rel” depict relative change in the respective outcome from intact to damaged (“_rel1”) and from damaged to repaired state (“_rel2). Variables ending in “_diff” depict absolute changes with the identical numbering scheme. SPSS data file.
(SAV)

S2 File Failure frequencies of the tested specimens during moderate axial compression, moderate axial torsion and ramp-to-failure. Weighted frequencies of the 3 groups during each testing step. SPSS data file.
(SAV)

S3 File Ultimate failure strength. Ultimate failure strength [N] of all samples surviving preceding moderate testing. SPSS data file.
(SAV)
